# Detection of Glycan Shedding in the Blood: New Class of Multiple Sclerosis Biomarkers?

**DOI:** 10.3389/fimmu.2018.01254

**Published:** 2018-06-04

**Authors:** Brian DellaValle, Alba Manresa-Arraut, Henrik Hasseldam, Allan Stensballe, Jørgen Rungby, Agnete Larsen, Casper Hempel

**Affiliations:** ^1^Department of Biomedicine/Pharmacology, Aarhus University, Aarhus, Denmark; ^2^Department of Clinical Microbiology, Copenhagen University Hospital, Copenhagen, Denmark; ^3^Department of Immunology and Microbiology, Faculty of Health and Medical Sciences, University of Copenhagen, Copenhagen, Denmark; ^4^Department of Biomedical Sciences, Faculty of Health, University of Copenhagen, Copenhagen, Denmark; ^5^Department of Health Science and Technology, Aalborg University, Aalborg, Denmark; ^6^Department of Endocrinology, Bispebjerg Hospital Copenhagen, Copenhagen, Denmark; ^7^Department of Micro- and Nanotechnology, Technical University of Denmark, Kongens Lyngby, Denmark

**Keywords:** glycocalyx, multiple sclerosis, precision medicine, biomarkers, glycosaminoglycans, proteoglycans, EAE, BBB

## Abstract

**Introduction:**

Multiple sclerosis (MS) is a devastating autoimmune disease, afflicting people in the prime of their lives. Presently, after initial clinical presentation, there are no reliable markers for whether a patient will develop MS, or whether their prognosis will be aggressive or relapsing–remitting. Furthermore, many MS patients do not respond to treatment. Thus, markers for diagnosis, prognosis, and treatment-responsiveness are lacking for a disease, where a precision medicine approach would be valuable. The glycocalyx (GLX) is the carbohydrate-rich outer surface of the blood vessel wall and is the first interaction between the blood and the vessel. We hypothesized that cleavage of the GLX may be an early stage predictor of immune attack, blood–brain barrier (BBB) breakdown, and disease severity in MS.

**Methods:**

Two experimental models of MS, experimental autoimmune encephalitis (EAE), were included in this study. EAE was induced in C57BL/6J mice and Lewis rats, which were monitored for weight loss and clinical presentation in comparison to healthy controls. Plasma samples were obtained longitudinally from mice until peak disease severity and at peak disease severity in rats. Soluble GLX-associated glycosaminoglycans (GAG) and proteoglycans (PG) were detected in plasma samples.

**Results:**

All animals receiving EAE emulsion developed fulminant EAE (100% penetrance). Increased plasma levels of chondroitin sulfate were detected before the onset of clinical symptoms and remained elevated at peak disease severity. Hyaluronic acid was increased at the height of the disease, whereas heparan sulfate was transiently increased during early stages only. By contrast, syndecans 1, 3, and 4 were detected in EAE samples as well as healthy controls, with no significant differences between the two groups.

**Discussion:**

In this study, we present data supporting the shedding of the GLX as a new class of biomarker for MS. In particular, soluble, sugar-based GLX components are associated with disease severity in two models of MS, molecules that would not be detected in proteomics-based screens of MS patient samples. Patient studies are presently underway.

## Introduction

Multiple sclerosis (MS) is a devastating autoimmune disease, often afflicting those in the prime of their lives. In the US alone, 200 new cases are diagnosed each week; however, current MS treatments are non-curative, side-effect prone, and expensive, highlighting the need for expanded treatment options (1, 2). Despite the advent of proteomics for assessing plasma biomarkers, treatment strategies are challenged by the lack of pathognomonic biomarkers predicting disease severity, early signs of new disease flares, and response-to-treatment (3, 4). For those diagnosed with clinically isolated syndrome, who have a 50% risk of progressing to MS, improved tools would evaluate their need for side-effect prone medical treatment. In this study, we utilize two variations of a well-described rodent model of MS, experimental autoimmune encephalitis (EAE) to identify a new and easily detectable class of potential biomarkers associated with disease debut and progression.

The glycocalyx (GLX) is a wide-spectrum term encompassing the diverse, carbohydrate-rich outer surface of the majority of cells in the body, including the luminal endothelium (5). Since this layer is the first interaction between the blood and the vessel wall, both throughout the body and specifically at the blood–brain barrier (BBB), we hypothesized that shedding of the GLX may be an early stage predictor of immune attack, BBB breakdown, disease severity, and treatment efficacy. Indeed, plasma detection of GLX shedding has been shown to be relevant for vascular permeability and overall disease progression for other inflammatory disease, such as sepsis, cerebral malaria, stroke, and trauma (6–11).

Due in part to an underestimation on its size, the biological and pathophysiological importance of GLX has been largely overlooked. Moreover, in the search for plasma biomarkers, sugar-based, GLX components would not be detected in large-scale protein-based assays, such as mass spectrometry. Indeed, proteomics studies have identified a plethora of plasma biomarkers associated with EAE and MS yet, to our knowledge, there have been no studies focused on the shedding of the GLX, in particular, glycosaminoglycans (GAGs) and proteoglycans (PGs). Chondroitin sulfate (CS), heparan sulfate (HS), and hyaluronic acid (HA) are GAGs shed from the GLX earlier than their membrane-anchored PG ectodomains, and thus may represent an early stage biomarker for attack or severity (12). In this study, we assess a timeline of GLX shedding of specific GAGs and syndecan (Syn) PGs over the course of EAE progression. We present the first evidence of early shedding of GAGs at different time points before and after disease debut. This data suggest that GAG detection should be investigated further as a potential biomarker for attack and severity in MS and response-to-treatment and could be valuable for increased minimally invasive disease monitoring.

## Materials and Methods

### EAE Induction

#### Myelin Oligodendrocyte Glycoprotein (MOG)-Induced EAE in C57BL6 Mice

Female C57Bl/6 mice {Taconic, Denmark (DK) aged 17 weeks [22.8 ± 0.4 g; early adulthood (13)]} were housed under standard conditions. EAE was induced in C57Bl/6 mice by active immunization with myelin oligodendrocyte glycoprotein (MOG) 35–55 using the kit EK-2110 from Hooke labs (MA, USA), following the manufacturer’s protocol. Briefly, mice were injected subcutaneously (s.c.) at two flanks with 200 µg of MOG 35–55 emulsified in Complete Freund’s adjuvant (CFA; *N* = 9), or 100 µl of PBS in case of the control mice (*N* = 6). At 2 and 24 h post-immunization the mice were injected i.p. with 100 µl of 4 µg/ml pertussis toxin (PTX) or 100 µl PBS for the control mice. Mice were monitored daily for clinical signs of disease and assigned a disease score according to the EAE clinical scoring system devised by the Danish Animal Experiments Inspectorate (see below).

#### Myelin Basic Protein (MBP)-Induced EAE in Lewis Rats

Female Lewis rats (Charles River, Germany) aged 14 weeks (219 ± 1 g) were administered an emulsion consisting of: 100 µl complete Freund’s adjuvant (CFA; BD 263810, DK), 200 µg *Mycobacterium tuberculosis* H37Ra (BD, 231141, DK), 100 µg guinea pig myelin basic protein (MBP; Sigma-Aldrich, DK, M2295), and 100 µl 0.9% saline (14).

Directly after preparation, a total of 200 μl emulsion was administered intradermally to animals for EAE under isoflurane anesthesia at three sites at the base of the tail, totaling 200 µl in volume (*N* = 10). MBP-EAE and control rats (*N* = 8) were treated with a small volume of saline twice-daily (100 μl), in accordance with the design of another study in order to limit the use of experimental animals. Since this set of animals was used as controls for a separate therapeutic intervention study, we could not sample blood longitudinally. Therefore, we obtained only terminal plasma samples at peak disease severity.

Studies were conducted to minimize suffering and were approved by the Danish Animal Inspectorate (2015-15-0201-00647 and 2012-DY-2934-00001). Weight was monitored daily throughout the experiment.

#### Clinical Scoring

Clinical scoring was performed twice daily using the following scale relating to progressive degrees of paralysis: 0, no clinical signs of EAE; 1, abolished tail tone; 2, mild paresis of one or both hind legs; 3, moderate paresis of one or both hind legs; 4, severe paresis of one or both hind legs; 5, paresis of one or both hind legs and incipient paresis of one or both forelegs are deemed moribund. Animals were deemed terminally ill according to predefined humane endpoints designed in consultation with the Danish Animal Inspectorate: animals registering a clinical score of ≥4, or a ≥20% loss of initial body weight.

### Timeline Sampling (MOG-EAE)

Before induction and from day 3 post-induction of EAE-MOG, a small volume of blood was collected from the facial vein into EDTA-powdered tubes for longitudinal samples. Blood was spun at 4°C and plasma isolated, flash frozen in liquid nitrogen, and stored at −80°C for analyses.

At the termination of the experiment (defined as “peak EAE scoring”), whole blood was isolated under 2% isoflurane anesthesia from the orbital plexus (C57Bl/6; anticoagulant, EDTA) or transcardially (Lewis Rat; anticoagulant: citrate), spun at 4°C, and plasma isolated, flash frozen in liquid nitrogen, and stored at −80°C for analyses.

### Enzyme-Linked Immunosorbent Assay (ELISA)

Hyaluronic acid in plasma was quantified with a commercially available ELISA kit (Echelon Biosciences, K-1200, Roskilde, Denmark).

### Plasma Dot Blotting

Due to the small volumes of plasma available with serial sampling, dot blots were used to assess GLX markers longitudinally, similar to our previous work (10). Two microliters of plasma were dotted in duplicate on a cationic nitrocellulose membrane (Hybond N^+^, Amersham, GE Healthcare, Brondby, Denmark) and allowed to dry. The membrane was incubated for 60 min at room temperature in blocking buffer: 5% skim milk powder (Sigma-Aldrich) in tris-buffered saline (Tris-buffered saline) + 0.05% Tween20 [Tris-buffered saline (TBS-T); Sigma-Aldrich]. The membrane was incubated thereafter with primary antibodies at their respective dilutions overnight, at 4°C. Membranes were thereafter washed in TBS-T and incubated with secondary antibodies conjugated to horseradish peroxidase, diluted at respective dilutions in blocking buffer, and raised against the source of the primary for 60 min. Membranes were washed thoroughly with TBS-T and finally in TBS. Membranes were visualized with Supersignal West femto luminescent substrate and Chemidoc XRS CCD camera (Bio-Rad Laboratories). Chemiluminescence was quantified with densitometry after normalizing to background with ImageJ software. Membranes were thereafter stripped with Restore Stripping Buffer (ThermoScientific, 21509) for 10 min at room temperature, washed in TBS and stripping was confirmed with identical development protocol (Femto, CCD camera). Membranes were blocked again with blocking buffer and probed for different antigens of interest (Second probe).

#### Primary and Secondary Antibodies

First Probing: HS (1:1,000, 10E4, cat no H1890, US Biological, MA, USA), Syn-1 (1:750, 281-1, cat no 553712, BD Pharmingen, Brøndby, Denmark), Syn-4 (1:750, KY8/2, cat no 550350, BD Pharmingen), CD44 (1:200, DAKO, Glostrup, Denmark). Second probing: CS (1:1,000, CS-56, cat no C8035, Sigma-Aldrich, Brøndby, Denmark), syndecan-3 (1:1,000, cat no AF3539, R&D Systems, UK). HRP-conjugated secondary antibodies: anti-rabbit (1:2,000), anti-rat (1:4,000), and anti-mouse (1:3,000) (DAKO, Glostrup, Denmark). All syndecan antibodies detect the syndecan protein structure and not carbohydrates.

### Data Analysis

Data sets were tested for normality (Shapiro–Wilk) and equal variance before statistical analyses were performed. Weight and dot blot data (MOG-EAE) were assessed with two-way ANOVA or Student’s *t*-test (MBP-EAE), ELISA data were assessed with Student’s *t*-test, and clinical scoring (MOG-EAE) was tested with Wilcoxon signed-rank test to determine when the median clinical score was statistically above 0. A *p*-value of <0.05 was reported as statistically significantly different. Data are presented as mean ± SEM for normal data and median ± interquartile range for non-normal data. Longitudinal data from MOG-EAE are presented as normalized to day −1, the day before the EAE emulsion was administered.

## Results

### EAE Induction

**Figure 1:**
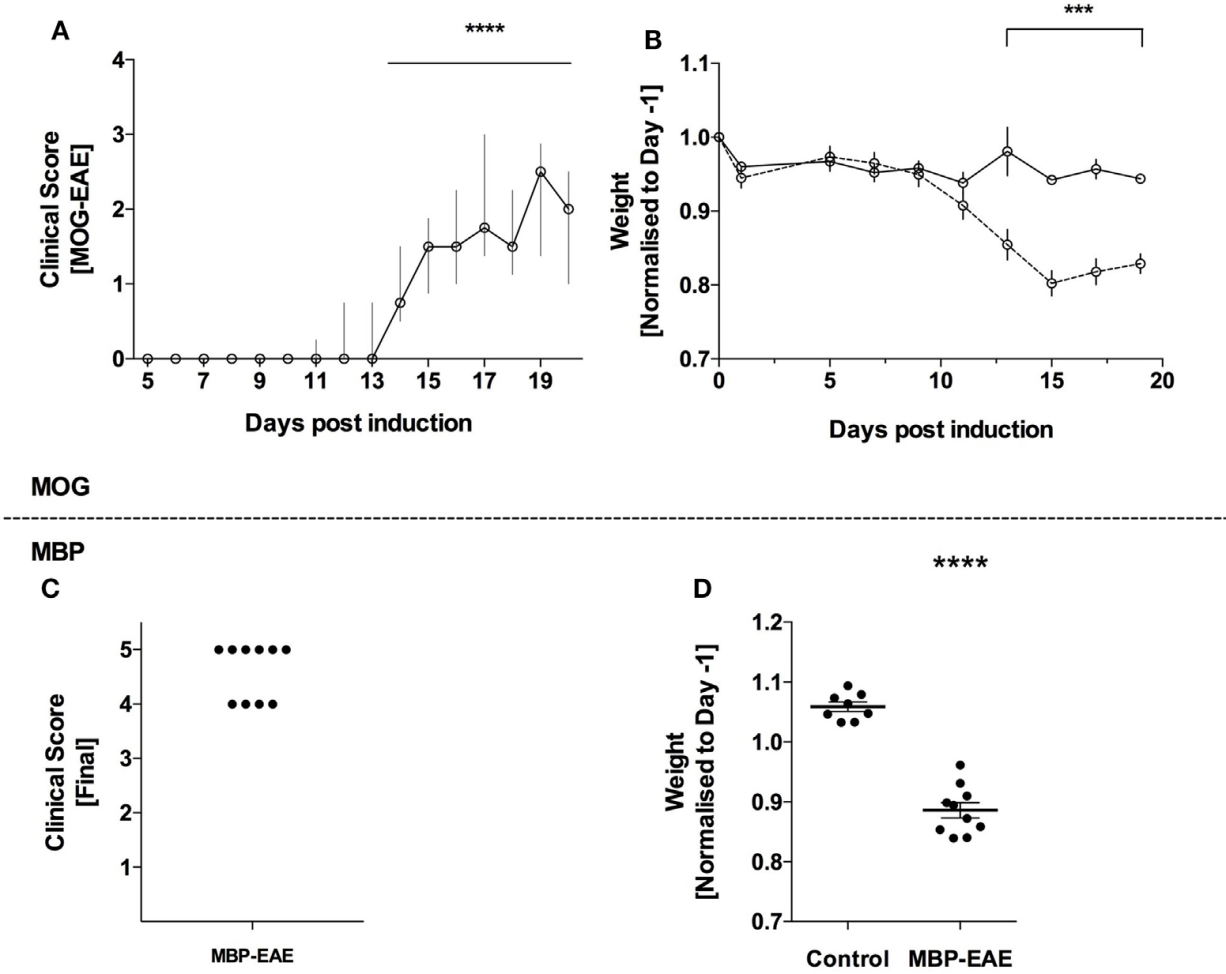
Full penetrance of experimental autoimmune encephalitis (EAE) induction in two models of multiple sclerosis. EAE was induced in C57Bl/6 mice with myelin oligodendrocyte glycoprotein (MOG-EAE) and in Lewis rats with myelin basic protein (MBP-EAE). **(A)** Clinical symptoms of increasing caudal–rostral CNS paralysis were significantly above zero from day 14 until peak disease. The experiment was terminated (day 20), coinciding with concomitant weight loss **(B)** in all mice that received MOG-EAE emulsion. MBP-EAE also induced clinical symptoms **(C)** and weight loss **(D)** in all rats receiving MBP-EAE emulsion. Data are presented as: **(A)** median with interquartile range; **(B)** average weight normalized to weight-before-emulsion (day −1); **(C)** dot blot of MBP-EAE rats included; **(D)** dot plot of weight normalized to weight-before-emulsion (day −1). Statistical differences were reported as *** or **** representing a *p*-value <0.001 and 0.0001, respectively, after testing for normality (Shaprio–Wilk) and equal variance and running the following statistical analysis: **(A)** Wilcoxon signed-rank test against the hypothetical value of 0; **(B)** two-way ANOVA; and **(D)** Student’s *t*-test. **(A,B)** *N* = control (6), MOG-EAE (9); **(C,D)** *N* = control (8) and MBP-EAE (10).

All animals receiving EAE emulsions developed clinical symptoms of paresis and experienced weight loss throughout the experiment representing a penetrance of 100% (Figure 1). As expected, an EAE-induced weight loss occurred 1–2 days prior to the additional signs of disease in both MOG-EAE (Figures 1A,B) and in MBP-EAE (Figures 1C,D). In MOG-EAE, clinical scoring was significantly above 0 from day 14 until termination (day 20) with a peak, median clinical score of 2.5 (day 19), and weight loss was significantly different from healthy controls from day 13.

### Detection of GLX Shedding in Plasma: GAGs Vary at Early- and Late-Stage of Disease

Due to constitutive turnover of GLX components, as expected, all soluble GLX markers tested were present in control plasma (15–17).

**Figure 2:**
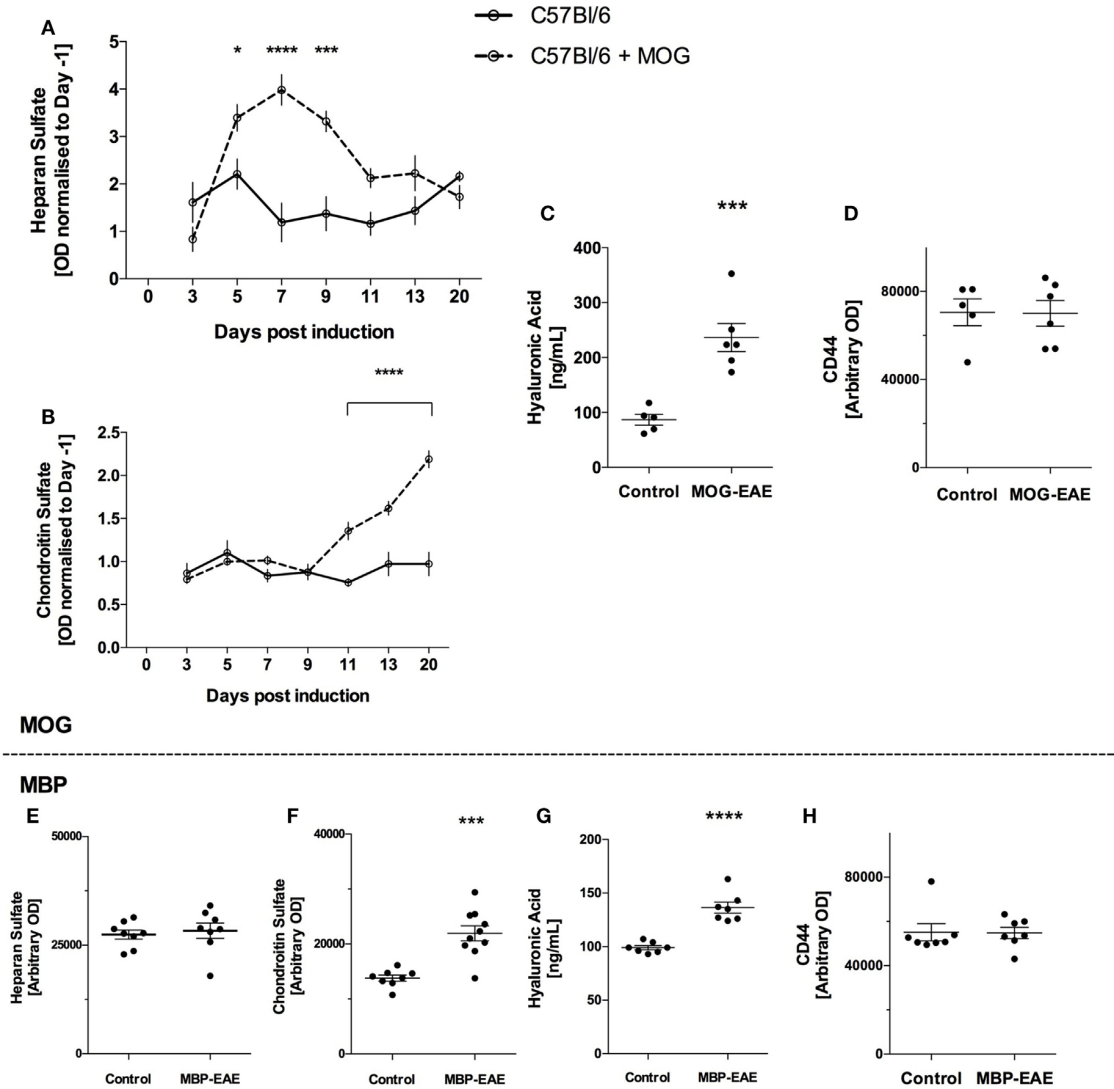
Sugar-based glycocalyx (GLX) markers significantly increased in plasma with disease course in myelin oligodendrocyte glycoprotein (MOG)-experimental autoimmune encephalitis (EAE) and are increased in late-stage myelin basic protein (MBP)-EAE. Sugar-based glycosaminoglycans heparan sulfate (HS), chondroitin sulfate (CS), and hyaluronic acid (HA) follow disease course in MOG- and MBP-EAE. **(A)** HS increases in the plasma at an early time point and returns to baseline are present manifest (Figure 1A). **(B)** Conversely, CS increases significantly in the plasma approximately ~2 days before clinical symptoms present in MOG-EAE. Detection of CS in the plasma proceeds to increase ~2-fold above controls at peak disease severity (day 20). **(C)** HA is ~2.5-fold higher in peak severity MOG-EAE when compared to controls. **(D)** CD44, an endothelial hyaluronate receptor did not vary between groups. Similar to MOG-EAE at peak disease severity, plasma from MBP-EAE at peak disease severity shows unchanged HS levels between diseased and control rats **(E)**, and significantly higher CS and HA levels when compared to controls **(F,G)**. CD44 **(H)** did not vary between groups. Data are presented as line graphs **(A,B)** normalized to pre-EAE levels (day 1) and **(C–H)** dot plots of control vs. EAE. Statistical significance is reported when *p*-value is <0.05 where *, ***, and **** refer to *p* < 0.05, 0.001, and 0.0001, respectively, after testing for normality (Shaprio–Wilk) and equal variance and running the following statistical analysis: **(A,B)** two-way ANOVA, **(C–E)** Student’s *t*-test.

In MOG-injected mice, HS levels were significantly above healthy controls from an early time point, day 5, and remained significantly above until day 11 (Figure 2A). Thereafter HS was no longer significantly above controls. Relating to Figure 1, HS peaked and returned to baseline before significant weight loss and clinical scoring occurred.

In contrast, CS levels were detected at similar levels to controls until day 11 where CS increased significantly (Figure 2B). This difference progressed steadily until the termination of the experiment, where CS levels in MOG-EAE mice were ~2-fold above controls. Control CS levels were relatively stable throughout the experiment. Relating to Figure 1, CS increased significantly ~2 days prior to weight loss and clinical scoring and steadily increased until peak disease activity.

Due to the volume required for ELISA, HA was only detected at termination of the experiment. As shown in Figure 2C, MOG-EAE resulted in significantly increased concentrations in plasma relative to controls (~2.5-fold from 86.8 ± 9.8 to 236.6 ± 25.7 ng/ml). Due the potential for binding of HA to the endothelial hyaluronate receptor CD44 (18), CD44 levels in plasma were also tested to assess whether potential CD44-HA complexes contribute to the observed difference. As shown in Figure 2D, CD44 did not vary in plasma from control and MOG-EAE mice (*p* = 0.95).

In late-stage MBP-EAE in Lewis rats, similar results were obtained for each GAG: HS levels were not different from controls (Figure 2E), whereas CS and HA levels were ~1.5-fold significantly above control levels (Figures 2F,G). CD44 was not different in plasma (*p* = 0.9, Figure 2H).

### Detection of GLX Shedding in Plasma: Proteoglycans

**Figure 3:**
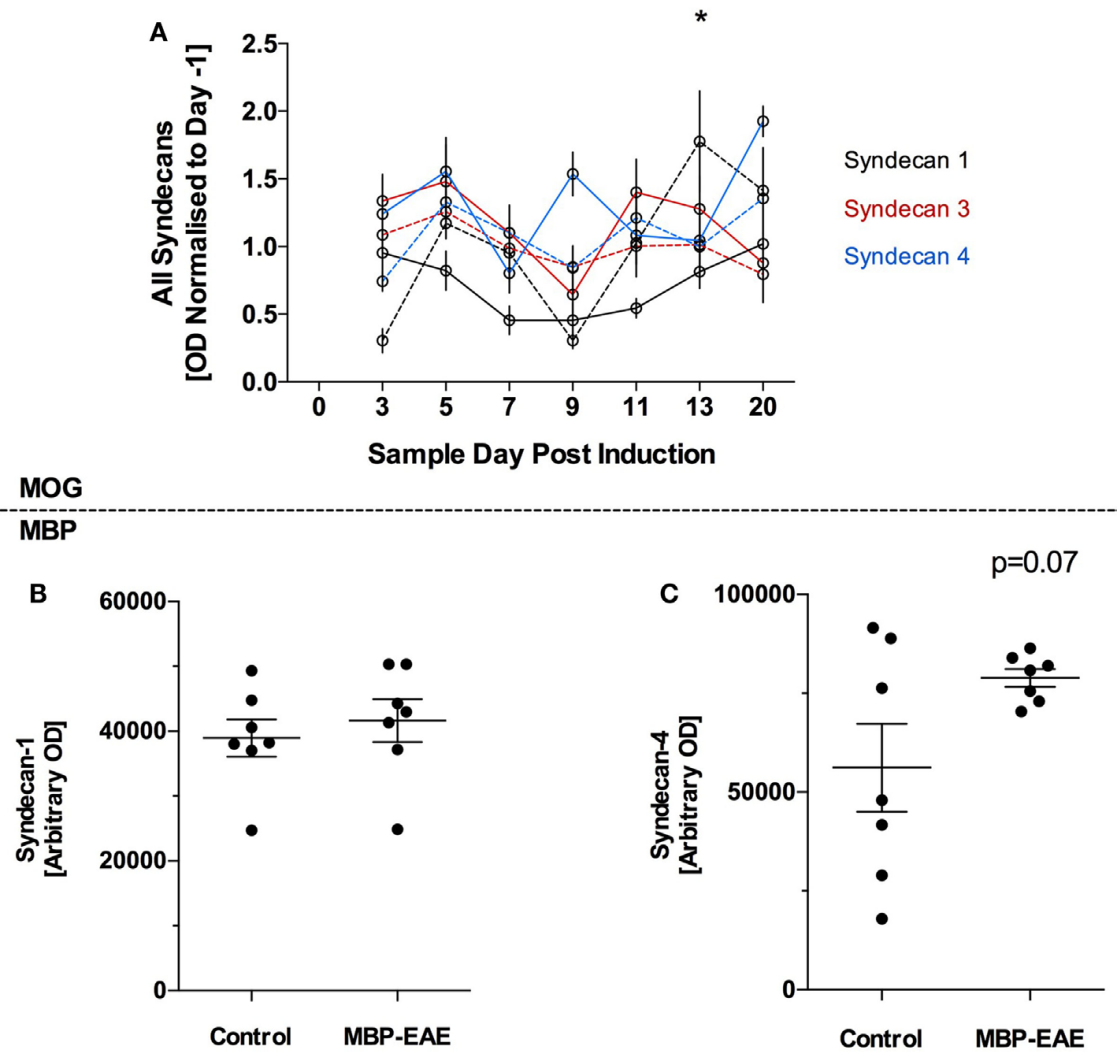
Protein-based glycocalyx (GLX) markers are largely unchanged in myelin oligodendrocyte glycoprotein (MOG)- and myelin basic protein (MBP)-experimental autoimmune encephalitis (EAE) in the initial stage of disease from induction until peak disease severity. GLX-based proteoglycans of the syndecan family were detected in the plasma of MOG- and MBP-EAE-induced rodents. In the MOG-EAE model **(A)**, levels of Syn-1 (black), Syn-2 (red), and Syn-4 (blue) were determined in controls (full line) and longitudinally until and at peak severity in disease (dotted line). In MBP-EAE, levels of Syn-1 **(B)** and Syn-4 **(C)** in controls and at disease peak severity were determined. **(A)** Inclusive of some day-to-day fluctuation, all three markers were relatively stable throughout the MOG-EAE disease course **(A)**. Nevertheless, Syn-1 was significantly increased in MOG-EAE at day 13. Syn-1 and -4 were not different in MBP-EAE at peak disease severity **(B,C)**. Syn-4 **(C)** tended to be higher than the control group but not significantly (*p* = 0.07), wherein the control group had a high internal variability. Data are presented as line graphs **(A)** normalized to pre-EAE levels (day −1) and **(B,C)** dot plots of control vs. EAE. Statistical significance is reported when *p*-value is <0.05, where * refers to *p* < 0.05 after testing for normality (Shapiro–Wilk) and equal variance and running the following statistical analysis: **(A)** two-way ANOVA, **(B,C)** Student’s *t*-test.

Syndecan-1, 3, and 4 were detected in plasma of MOG-EAE and control mice throughout the experiment (Figure 3) and all three markers were relatively stable throughout the MOG-EAE disease course, albeit inclusive of day-to-day fluctuation (Figure 3A). Syn-1 was significantly above controls at day 13; however, it was not significantly different at the termination of the experiment.

In late-stage MBP-EAE, a similar pattern was observed: no differences were detected between MBP-EAE and controls (Figures 3B,C), albeit a trend was detected in favor of higher shedding of Syn-4 in late-stage MBP-EAE (Figure 3C, *p* = 0.07).

## Discussion

With this study, we propose that the shedding of the polysaccharide-rich GLX may be a useful marker for disease activity in MS. This is based on the first evidence that distinct, soluble GAGs of the GLX are responsive to experimental myelin-based autoimmunity in the plasma of rodents. Rodent EAE models have many pathophysiological similarities to human MS and have contributed to the understanding of MS pathology and the development of therapeutic interventions (19).

In the search of pathognomonic biomarkers of MS, a plethora of candidate molecules have been proposed, generated through both targeted studies and large-scale proteomic- and transcriptomic studies. Potential markers include chemokines, cytokines, cholesterols, growth factors, microRNAs, brain-derived proteins, proteases, autoantibodies, and anti-viral antibodies (3, 4, 20, 21). The present golden standard for MS diagnosis remains an assessment of expanded disability status scale, brain volume/lesion size MRI, and, in some facilities, cerebrospinal oligoclonal/immunoglobulin index (2, 4). Interestingly, the distinct glycan molecules in this study would not be detected in proteomic screens of plasma, and thus represents a new class of candidate biomarkers readily available through simple antibody-based testing of small volumes of plasma or serum.

### Glycans in the Peripheral Blood: Promising New Class of Biomarker in MS?

Longitudinally, MOG-EAE showed differing shedding curves with respect to the distinct glycans studied (Figure 2A–C). HS shed significantly higher than controls at an early phase, before presentation of clinical symptoms, and returned to baseline by day 11 as the clinical symptoms began to present. Contrasting, CS shedding closely followed the development of clinical symptoms and peaked at study termination. HA was significantly higher in MOG-EAE than controls at the peak of disease. This difference was not related to CD44 levels in the plasma and can, therefore, be attributed to unbound plasma HA (18). Furthermore, this indicates that HS and CS/HA shedding reflect different parts of the disease pathology. As the EAE model system is known to induce a T-cell response to a myelin protein, it is interesting to further investigate if animals exposed solely to the adjunctive elements, i.e., the CFA/PTX, would indeed mimic the early HS response. Nevertheless, CS and HA are indeed the most EAE-specific elements of the GAGs.

### Glycans in the Peripheral Blood: Increased Sheddase Activity?

Specific cleavage of HS is mediated by different types of endogenous heparanases: one located in the endothelium and others located in circulating immune cells and platelets (22, 23). We suggest that heparanase activity is increased as response to CFA, since PTX was previously shown to inhibit G-protein coupled receptor-mediated shedding (12). Thus, with CFA being immunostimulatory it is likely that the injection leads to heparanase secretion from leukocytes. CFA/PTX does not seem to stimulate other sheddases, since the other markers assessed were unaffected by this injection. Nevertheless, it could be expected that plasma HS would increase during a later phase of EAE where glial expression of heparanase is increased (24). Since we followed the disease course only until peak disease, this effect may emerge in the peripheral blood after a longer time interval.

Heparan sulfate is the most abundantly present GAG in the endothelial GLX followed by HA and CS (5). In contrast, CS levels in tissues are generally higher than HS. Intriguingly, increased plasma levels of CS coincided with clinical symptoms. Moreover, HA levels in plasma were substantially increased. The enzyme hyaluronidase is found in platelets and leukocytes, has specificity for both of these GAGs, and thus, may be involved in the shedding we observe (25, 26). Furthermore, increased plasma levels of HA could also be a result of the inflammatory conditions present in EAE which stimulates HA synthesis (27), however, endothelial coverage decreases overall, suggesting that HA turnover under some inflammatory conditions is not fully compensated by synthesis. Both CS and HA were previously shown to be involved in CNS injury (28). In addition to endothelial origin, the source of these markers in the blood could also be the CNS proper, due to an increased BBB permeability. PTX injection has been shown to induce a transient BBB opening, whereas an EAE-induced opening coincides with leukocyte entering the nervous tissue and initiation of the clinical phase thereafter (29). This is reflected in the CS response, where increased levels in the MOG-EAE animals were seen 2–3 days before clinical disease debut.

Beyond their compositional differences, GAGs are produced in different ways: HS and CS are produced in the Golgi compartment, polymerized and transported to the cell surface anchored to its respective proteoglycan; whereas HA is synthesized at the cell surface and not covalently attached to any cell surface proteins (5, 28). This has importance for interpretation of the shedding. Detecting HA in the plasma suggest hyaluronidase activity, while detection of CS could be the result of both hyaluronidase activity and protease activity. Therefore, as we do not observe an increase in the endothelial-specific PGs, we interpret this to be a result of increased hyaluronidase activity in the cerebrovascular compartment and/or in the cerebral parenchyma proper (25, 28, 29).

### Glycans in the Peripheral Blood: MOG-EAE vs. MBP-EAE

Glycosaminoglycans levels at the peak-stage of EAE were similar in result and magnitude in both rodent models: MOG-EAE increased CS and HA by 2- to 2.5-fold, respectively with no change in HS, and MBP-EAE increased CS and HA by 1.5-fold with no change in HS, compared to respective controls. However, MBP-EAE rats show higher disease severity than MOG-EAE mice [~4.5 vs. 2.5, respectively (Figure 1)].

### Proteoglycans in the Peripheral Blood

In the disease phase of this study, shedding of the GLX ectodomains of Syn-1, 3, and 4 seemed to be unaffected by inflammation, endothelial activation, and nervous tissue damage. Our data suggest that loss of PG itself is not extensive at these time points. Since this study involves only the induction phase and first clinical peak of EAE we cannot rule out that PG ectodomains may begin to shed above control levels at a more advanced time after EAE induction.

### GAGs and Pathogenesis of EAE

Glycosaminoglycans play multiple important roles in EAE pathogenesis. Initial leukocyte rolling and infiltration into the CNS can be reduced by removing GAGs from the vasculature (30, 31). This is further substantiated by i.v. injections of soluble GAGs that serve as an extra reservoir of binding partners for leukocytes, resulting in decreased neuroimmunity (32–34). Moreover, GAGs play a key role in controlling neuroinflammation, where HA accumulation has been shown to increase in demyelinated lesions and impair remyelination (35) and pharmacological removal of HA reduces spinal cord demyelination (30). Also, CS accumulation has been shown to impair remyelination (36). Furthermore, GAGs can activate and recruit immune cells and are thought to be endogenous signals for damage (37, 38). Therefore, the use as biomarkers for CNS inflammation, and possibly MS, is highly promising, further underlined by this study. Interestingly, others have shown that Syn-1 knockout animals have a worse prognosis in MOG-EAE and that Syn-1 is increased in the cerebrospinal fluid of mice at peak EAE (39). Moreover, it has been reported from polymorphism studies that the gene for another PG, glypican-5, is associated with MS development (40) and with responders to interferon-beta treatment (41) albeit, the pathogenic significance of this finding is unclear.

## Conclusion

We present evidence of glycan shedding in experimental MS and a longitudinal course of shedding that appears relevant as a potential new class of biomarker for autoimmune inflammation. Ideally, a biomarker would be absent in healthy individuals and present in diseased patients. Indeed, the power of glycan shedding to detect difference in disease vs. non-diseased animals from two models of MS is a strong indicator that further, detailed studies of these molecules in patients are warranted. We are presently assessing the translational relevance of this finding in MS patients with the ultimate goal of improving disease monitoring and predictability, and providing a reliable complementary diagnostic tool for treatment response.

## Ethics Statement

Female C57Bl/6 mice (Taconic, Denmark) aged 17 weeks (22.8 g) and female Lewis rats (Charles River, Germany) aged 14 weeks weighing (219 g) were housed under standard conditions. Studies were conducted to minimize suffering and were approved by the Danish Animal Inspectorate (2015-15-0201-00647 and 2012-DY-2934-00001). Weight was monitored daily throughout the experiment. Animals were monitored daily for clinical signs of disease and assigned a disease score according to the EAE clinical scoring system devised by the Danish Animal Experiments Inspectorate. Animals were deemed terminally ill according to predefined humane endpoints designed in consultation with the Danish Animal Inspectorate: animals registering a clinical score of ≥4, or a ≥20% loss of initial body weight.

## Author Contributions

Designed, conducted, analysis, interpretation, and drafted manuscript: BV. Designed, analysis, interpretation, and drafted manuscript: AL and CH. Designed, conducted, and drafted manuscript: AM. Analysis, interpretation, and drafted manuscript: HH, AS, and JR.

## Conflict of Interest Statement

Aarhus University and Technical University of Denmark are owners of a patent related to this work wherein BV, AM-L, and CH are inventors.

## Abbreviations

BBB, blood–brain barrier; CFA, complete freund’s adjuvant; CS, chondroitin sulfate; DK, Denmark; EAE, experimental autoimmune encephalitis; ELISA, enzyme-linked immunosorbent assay; GAG, glycosaminoglycans; GLX, glycocalyx; HA, hyaluronic acid; HS, heparan sulfate; MBP, myelin basic protein; MOG, myelin oligodendrocyte glycoprotein; MS, multiple sclerosis; PG, proteoglycans; PTX, pertussis toxin; Syn, syndecan; TBS-T, Tris-buffered saline.
